# Functional Neural Networks in Human Brain Organoids

**DOI:** 10.34133/bmef.0065

**Published:** 2024-09-23

**Authors:** Longjun Gu, Hongwei Cai, Lei Chen, Mingxia Gu, Jason Tchieu, Feng Guo

**Affiliations:** ^1^ Department of Intelligent Systems Engineering, Indiana University Bloomington, Bloomington, IN 47405, USA.; ^2^ Center for Stem Cell and Organoid Medicine (CuSTOM), Division of Pulmonary Biology, Division of Developmental Biology, Cincinnati Children’s Hospital Medical Center, Cincinnati, OH 45229, USA.; ^3^ University of Cincinnati School of Medicine, Cincinnati, OH 45229, USA.

## Abstract

Human brain organoids are 3-dimensional brain-like tissues derived from human pluripotent stem cells and hold promising potential for modeling neurological, psychiatric, and developmental disorders. While the molecular and cellular aspects of human brain organoids have been intensively studied, their functional properties such as organoid neural networks (ONNs) are largely understudied. Here, we summarize recent research advances in understanding, characterization, and application of functional ONNs in human brain organoids. We first discuss the formation of ONNs and follow up with characterization strategies including microelectrode array (MEA) technology and calcium imaging. Moreover, we highlight recent studies utilizing ONNs to investigate neurological diseases such as Rett syndrome and Alzheimer’s disease. Finally, we provide our perspectives on the future challenges and opportunities for using ONNs in basic research and translational applications.

## Introduction

In the human brain, billions of neurons are interconnected by synapses, forming intricate brain neural networks (BNNs). These neural networks are responsible for various physiological functions, from basic reflexes to advanced cognitive processes [1–3]. For instance, BNNs play a crucial role in encoding, storing, and retrieving memories, and thus are the foundation of learning and memory [4]. Unfortunately, BNNs are particularly vulnerable. They can undergo notable changes in various neurological diseases, ranging from neurodegenerative diseases like Alzheimer’s and Parkinson’s to neuropsychiatric disorders such as autism and schizophrenia [5,6]. These changes in BNNs can occur at either the single-cell or circuit level, resulting in their dysfunction. Despite pioneering efforts, challenges remain in understanding the physiological functions and pathological performance of BNNs.

So far, several models of BNNs are used to study complex brain functions and disease mechanisms. These models are particularly valuable to understand the neurological, psychiatric, and developmental disorders that show evident network abnormalities without accompanying overt anatomical changes. Traditional 2-dimensional (2D) in vitro neuron cultures are homogeneous and fail to recapitulate the structure and 3D microenvironment of the BNNs in vivo. While animal models have been instrumental in neuroanatomical and behavioral studies, the mouse, the most widely used animal model, has some marked differences in brain development compared to humans [7,8]. The human brain is substantially larger, containing approximately 86 billion neurons compared with 71 million in mice [8–10]. In addition, the outer radial glial progenitors (oRGs), which play a crucial role in the radial and tangential expansion of the cerebral cortex, are more pronounced in primates’ outer subventricular zone (OSVZ) compared to rodents. As a result, a well-defined OSVZ is recognizable in the developing human brain [11]. Moreover, the gene expression profiles for genes involved in cortical development have marked differences between humans and mice [12,13]. Human brain organoids are self-organized tissues derived from human pluripotent stem cells (hPSCs) [14,15]. This emerging model contains key cell types of the embryonic human brain, such as neural progenitors, neurons, and glial cells. Meanwhile, they can faithfully recapitulate certain structural aspects such as the formation of a ventricular- and subventricular-like zone, where early neurogenesis occurs. More importantly, human brain organoids can be derived from patient-specific induced PSCs, providing a window into early human brain development, and enabling the study of neurological disease in individuals.

**Fig. 1.**
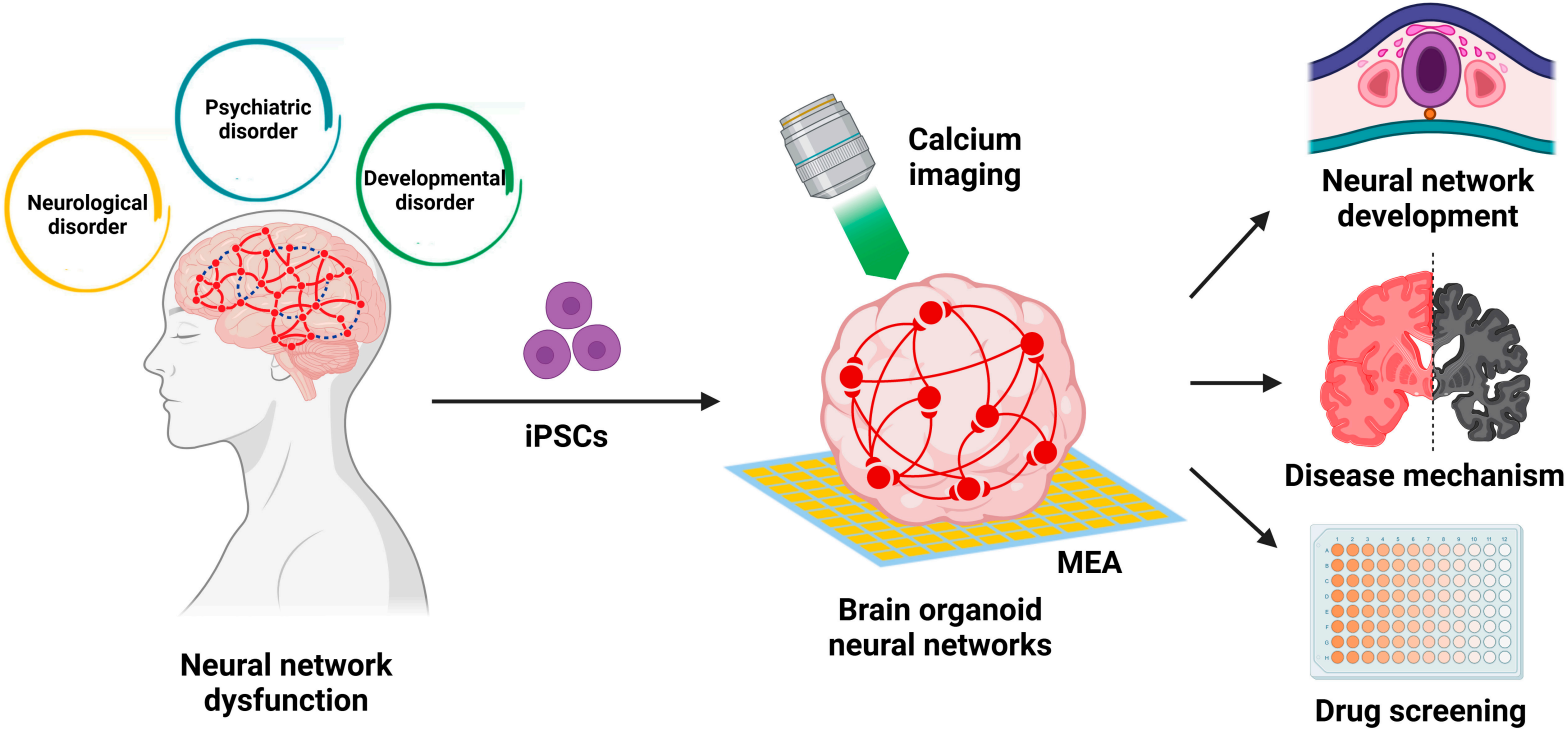
Brain organoid neural networks. Calcium imaging and MEA recording are 2 mainstream strategies for neural network detection in brain organoids. Brain organoid neural networks offer a versatile tool for understanding brain neural network development, disease mechanisms, and drug screening.

Here, we review the recent research advances on understanding, characterization, and application of functional brain organoid neural networks (ONNs). We first introduce the formation of ONNs. Then, we discuss the detection strategies of ONNs and highlight recent studies that leverage ONNs for modeling neurological, psychiatric, and developmental disorders. Finally, we provide perspectives on using the ONNs as a versatile tool for advancing the understanding of BNN development and disease pathogenesis, with broad implications for basic neuroscience research and therapeutic development (Fig. 1).

## Formation of Functional Brain Organoid Neural Networks

In humans, the formation of BNNs begins during embryonic development and continues throughout life. In the cerebral cortex, the differentiation of radial glial-like neural stem cells is responsible for the canonical 6-layer cytoarchitecture found in the dorsal forebrain and includes the differentiation of several neuron subtypes as well as glia [16,17]. Intermediate progenitor cells are immediate descendants of radial glial-like neural stem cells. These cells reside in the subventricular zone (SVZ) and intermediate zone (IZ) of the developing brain. Their key function is to amplify neuronal production through mitosis. Inhibitory neurons originate from the ganglionic eminences, and some migrate tangentially toward the dorsal cortex to establish inhibitory circuits crucial for proper network function [18,19]. As soon as neurons approach their preprogrammed destinations, they extend axons and dendrites through growth cones with highly motile and responsive properties and form synaptic connections [20,21]. During this phase, the presynaptic neuron releases neurotransmitters across the synaptic cleft, and the postsynaptic neuron forms corresponding receptor sites, establishing functional synapses. As the brain develops, an overproduction of synapses occurs, followed by activity-dependent synaptic pruning, where less active synapses are eliminated to refine neural circuits. This synaptic plasticity ensures the strengthening of frequently used connections, facilitating efficient communication within the network. Ultimately, through these dynamic processes, neurons integrate into complex, functional neural networks. Excitatory neurons activate postsynaptic neurons by releasing neurotransmitters such as glutamate. In contrast, inhibitory neurons release γ-aminobutyric acid (GABA) to maintain the balance between excitation and inhibition in neural circuits. This balance is essential for preventing hyperexcitability and ensuring the stability of BNNs during development. Except for neurons, glial cells also play supportive roles in maintaining BNNs. Astrocytes help regulate the extracellular environment, provide metabolic support, and modulate synaptic transmission. Oligodendrocytes are responsible for the myelination of axon bundles, which enhances the speed and electrical signal transmission.

### Unguided brain organoid development method

**Fig. 2.**
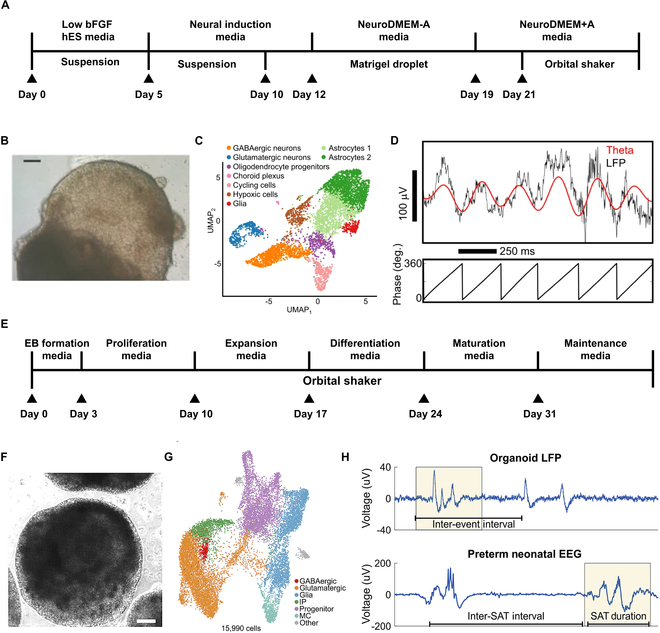
The formation of neural networks in brain organoids. (A) Schematic of the unguided development method for generating cerebral organoids. (B) Bright-field image of the cerebral organoid. Scale bar, 200 μm [26]. (C) Single-cell transcriptomes of human cerebral organoids (*n* = 3 cerebral organoids, 5,680 cells) colored by 9 cell clusters: orange as GABAergic neurons, blue as glutamatergic neurons, red as glia cells, green as astrocytes, purple as oligodendrocyte progenitors, light pink as cycling cells, dark pink as choroid plexus, and brown as hypoxic cells [31]. (D) Top: Local field potential (<500 Hz, black line) and the 4- to 8-Hz theta-filtered band (red line). Bottom: Phase of the theta oscillation [31]. (E) Schematic of the guided development method for generating cortical organoids. (F) Bright-field image of cortical organoid. Scale bar, 200 μm [40]. (G) Single-cell transcriptomes of human cerebral organoids (15,990 cells) colored by 7 cell clusters: red as GABAergic neurons, orange as glutamatergic neurons, blue as glia cells, green as intermediate progenitors, purple as progenitors, green blue as mitotic cells, and gray as others [40]. (H) Local field potential trace from cortical organoid. Comparable events between periods of quiescence (discontinuous network dynamics) are shown in human preterm neonate EEG at 35 weeks gestational age. SAT, spontaneous activity transient [40].

Remarkably, human brain organoids can recapitulate some certain developmental trajectories and contain these key cell types to establish functional connections and networks [22]. Several protocols have been well developed to generate human brain organoids with functional ONNs [23–25]. The unguided development method induces hPSCs to differentiate into cerebral organoids through intrinsic signals instead of providing extra patterning and growth factors [26–29]. This protocol relies on the generation of embryoid bodies (EBs), which are induced into the neuroectoderm. Then, the EBs are embedded into Matrigel and transferred to a bioreactor to promote neuroepithelium expansion, neural differentiation, and organoid maturation (Fig. 2A and B). With minimal external influence during the differentiation process, stem cells tend to self-organize and differentiate into discrete brain regions, such as the forebrain, midbrain, and hindbrain. Single-cell sequencing reveals the presence of the key cell types within these cerebral organoids like excitatory neurons, inhibitory neurons, and astrocytes (Fig. 2C). These organoids exhibit spontaneous electrophysiological activity in both stable firing and burst firing patterns [30]. The synchrony and phase relationship between theta oscillations and local neuronal firing indicates that rhythmic activity originates from local ONNs (Fig. 2D) [31]. Although unguided cerebral organoids provide unique opportunities to investigate the connections between different brain regions, high variability as well as the heterogeneity of organoids are stumbling blocks for quantitative studies.

### Guided brain organoid development method

The guided protocols use small molecules and growth factors throughout the majority of the differentiation process to induce neuroectodermal cells into organoids representative of specific brain regions, such as cortical [32–34], midbrain [35], and hippocampus [36]. These guided organoids have relatively consistent cell composition and proportion, reducing the variation across different batches and cell lines. For cortical organoid generation, hPSCs are first induced to form the neuroectoderm by suppressing the transforming growth factor-β (TGF-β) and bone morphogenetic protein (BMP) signaling pathways [37]. Then, epidermal growth factor (EGF) and basic fibroblast growth factor (bFGF) are used to maintain the proliferative capacity of neural precursors [38]. Brain-derived neurotrophic factor (BDNF), glial cell line-derived neurotrophic factor (GDNF), and neurotrophin-3 (NT3) are subsequently introduced to accelerate the differentiation and maturation of the cortical organoids (Fig. 2E and F) [39]. In the generated cortical organoids, 5 major cell types (excitatory neurons, inhibitory neurons, glial cells, progenitors, and intermediate progenitors) are identified based on specific marker gene expression (Fig. 2G). Neural networks in cortical organoids continually mature with the enhancement of electrophysiological activity, as quantified by firing rate, burst frequency, and synchrony index. Interestingly, periodic oscillatory network activities are observed in 8-month-old organoids. Although these neural network activities do not recapitulate the full temporal complexity in adults, synchronous network events exhibit characteristics comparable to those seen in preterm neonatal electroencephalography (EEG) (Fig. 2H). The timing features include spontaneous activity transients (SATs) per hour, and 95% of inter-SAT duration exhibits a high degree of similarity between cortical organoids and preterm infants [40].

To further integrate organoids into sensory and motivation-related circuits, the cortical organoids are transplanted into the somatosensory cortex of newborn athymic rats [41]. The somatosensory cortex processes tactile sensations such as whisker movement. The organoids increased 9-fold in volume over 3 months after transplantation. Neurons in transplanted cortical organoids exhibit more complex morphological features, synaptic connections, and intrinsic membrane properties compared to their in vitro counterparts. Interestingly, transplanted cortical organoids can be activated by electrical stimulation and by stimulating the rat’s whiskers, indicating that the organoids are receiving meaningful sensory input. Additionally, researchers can activate human neurons within the transplanted organoid to influence the rat’s reward-seeking behavior. These findings suggest that the transplanted organoid functionally integrates into specific brain pathways. In another study, the cortical organoids are transplanted into the retrosplenial cortex of adult mice [42]. By placing a transparent microelectrode array (MEA) on top of the transplanted organoids, neural activity electrically from both the implanted organoid and the surrounding host cortex can be recorded in real-time. Using 2-photon imaging, mouse blood vessels that grew into the organoid are observed, providing necessary nutrients and oxygen to the implant. The detected electrical activity in the organoids indicates that the transplanted organoids respond to visual stimulus in the same way as surrounding tissue. In addition, graphene recordings reveal increases in the power of gamma oscillations and phase locking of spikes from organoids to slow oscillations from the mouse visual cortex. These results suggest that the organoids have formed synaptic connections with surrounding cortex tissue and receive functional input from the mouse brain.

In summary, the human brain organoids produced by these 2 different differentiation protocols have some similarities: (a) both contain critical cell types necessary for the generation of functional ONNs; (b) both can support self-organized patterns of activity. The choice between unguided and guided brain organoid development methods will depend on specific applications. Unguided brain organoids contain multiple brain regions, thus suitable for investigating ONN interactions between different brain regions. In contrast, guided brain organoids are better for exploring the physiological functions and pathological changes of ONNs in specific brain regions.

## Functional Characterization of Brain Organoid Neural Networks

Neuroelectrical activity is essential for neuronal communication and information processing in BNNs. The basis of neuroelectrical activity lies in the dynamic changes of ions across the neuronal membrane. When a neuron receives a signal or stimulus, it can depolarize or hyperpolarize. Depolarization occurs when there is an influx of positively charged ions into the neuron, leading to a reversal of the membrane potential and triggering an action potential that propagates along the axon. Based on this principle, the physiological status and pathological changes of ONNs in the brain organoids can be evaluated by monitoring the concentration of ions inside the neurons or the extracellular action potential. Currently, several methods have been developed for the functional characterization of ONNs.

### Multiphoton microscopy-based calcium indicator imaging

**Fig. 3.**
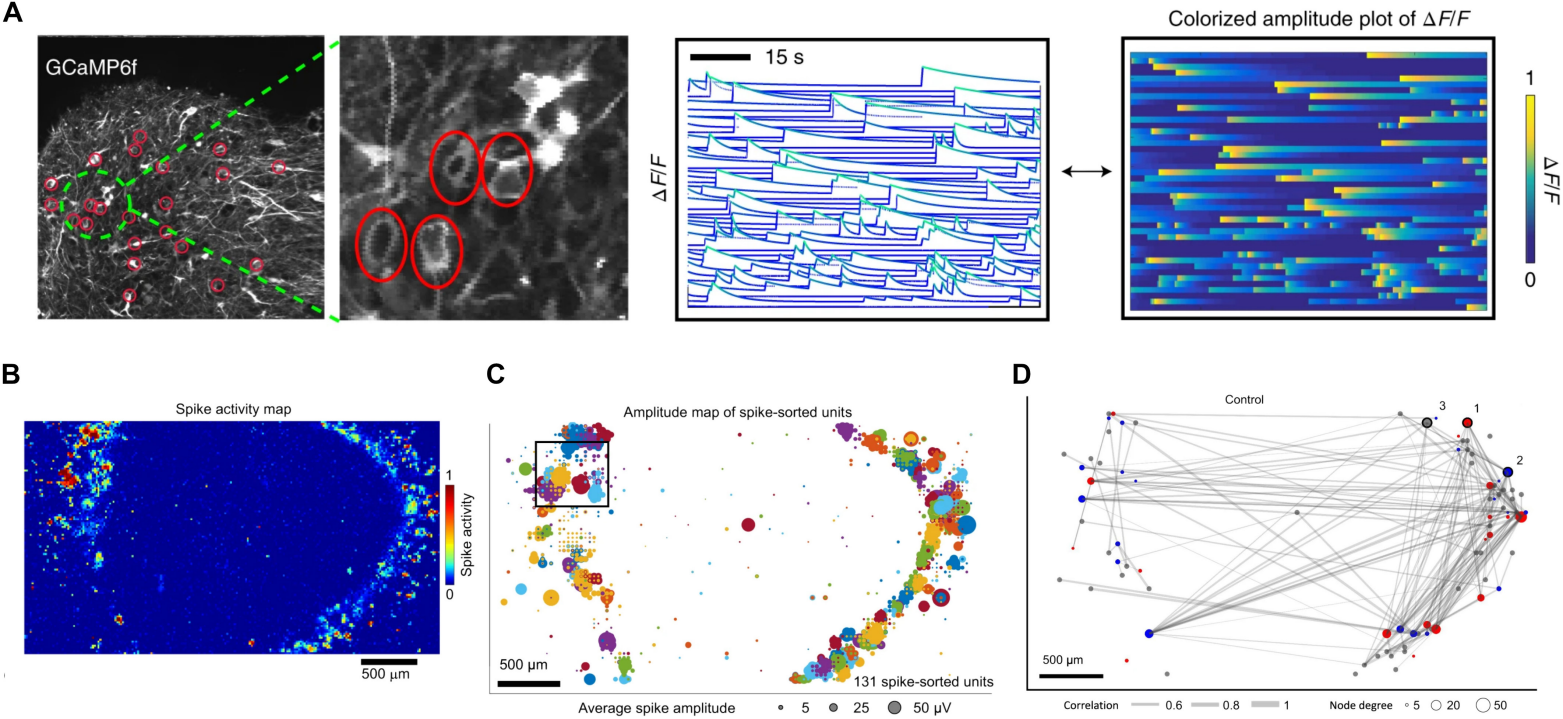
Strategies for neural network detection in brain organoids. (A) Two-photon microscopy imaging of cerebral cortex–ganglionic eminence fusion organoid and the activity profile shown as normalized Δ*F*/*F* values. Each line means an individual neuron and representation of the same data as a colorized amplitude plot [45]. (B) Spike activity map of a cerebral organoid slice positioned on a high-density CMOS microelectrode array to survey the electrical activity across the entire organoid. The color scale indicates the normalized number of detected spikes. Scale bar, 500 μm [31]. (C) Spatial map of the average spike amplitude (bubble size) from single-unit activity measured simultaneously across the CMOS array from the top 1,020 electrode sites based on activity. Single-unit electrode clusters are plotted using the same color (the same colors are repeated for different units) [31]. (D) Functional connectivity map showing the temporal correlation strength between spike trains of neurons (edge thickness in gray). Sorted by directionality, the in-degree and out-degree were computed per unit, defined as predominately incoming or outgoing edges, respectively, and designated “receiver” (blue) nodes and “sender” (red) nodes. All other nodes were labeled “brokers” (gray with a fixed size not indicative of node degree) [31].

Multiphoton microscopy-based calcium indicator imaging is an advanced technique for visualizing neuronal activity in brain organoids with high spatial resolution. Two-photon imaging has an outstanding advantage in deep tissue observation based on nonlinear excitation with longer wavelengths. As a result, the activity of neurons within and on the surface of brain organoids (up to 800 μm) can be simultaneously observed. This technique leverages the properties of calcium fluorescent indicators, which emit fluorescence upon binding to calcium ions [43]. Calcium ions play a crucial role in neuronal activity, with their intracellular concentration increasing during action potentials and synaptic events. Brain organoids are labeled with chemical calcium indicators (e.g., Fluo-4 and Fura-2) or introduced with genetically encoded calcium indicators (e.g., GCaMP and RCaMP), and subsequently placed under the two-photon microscope for calcium imaging [44,45]. Spontaneous calcium activity can be monitored in real-time through transient fluorescence activity (Fig. 3A). Regions of interest in brain organoids were selected and calculated Δ*F*/*F* values, of which Δ*F* represents the change in fluorescence intensity over time relative to the baseline fluorescence intensity and *F* represents the raw fluorescence intensity value. In consequence, the functional connectivity map of the organoids can be visualized by employing a graph-based representation of the connectivity matrix [46]. Using this technique, up to 1,820 cells within an ONN can be detected simultaneously for their calcium transients and further distinguish the synchronized and non-synchronized clusters. The calcium transients of individual neurons tend to be synchronized with the maturity of ONNs [44]. However, the synchronized burst can be blocked by introducing GABA_A_ receptor antagonists such as bicuculline methiodide or gabazine into the brain organoids [45,47].

### Microelectrode array (MEA) recording

The MEA system uses a range of tiny electrodes to simultaneously detect extracellular electrical signals from cells [42,48]. The MEA measurements provide high temporal resolution and the ability to monitor large populations of neurons over extended periods. Therefore, this technique is more suitable for studying the dynamic development of ONNs in long-term cultured brain organoids, which have been described previously [40]. In addition to the above advantages, the number and spatial resolution of detectable neurons remain as challenges for this method: (a) Current MEA chips have relatively small numbers of microelectrodes (<100 microelectrodes per well), and large inter-electrode spacing can only monitor population activity instead of single neuronal activity, missing signals from the majority of neurons [49]; (b) only neurons at the surface of the organoid that adhere to microelectrodes can be detected [50,51].

To improve spatial resolution, complementary metal oxide semiconductors (CMOSs) are combined with the MEA to read the extracellular field potentials of single neurons in a high-resolution manner [52,53]. This MaxWell Biosystems fabricates the high-density CMOS-MEA systems with 26,400 platinum microelectrodes, of which up to 1,024 microelectrodes can be recorded simultaneously. The size of each microelectrode (9.3 × 5.3 μm^2^) is almost at a single neuron scale. Meanwhile, the entire sensing area is as large as 3.85 × 2.10 mm^2^, which is suitable for investigating the neural activity of large-scale ONNs [47]. Human brain organoids are sectioned into 500-μm-thick tissue slices to expose the deep structure and then adhere to MEA. Organoid slices can potentially improve cell growth and nutrient distribution within the tissue and promote axon outgrowth. A spatial map of the extracellular action potential activity of the organoid slice can be obtained through an automated scan of contiguous tiled blocks (Fig. 3B) [31]. The top 1,024 microelectrodes are selected based on their spiking frequency. These data could be further used to generate the amplitude map of spike-sorted units by employing the spike-sorting algorithm Kilosort2 (Fig. 3C). Consequently, temporal correlation strength between spike trains of neurons was quantified by using the spike time tiling coefficient (STTC) (Fig. 3D) [54].

### Magnetic resonance imaging

Magnetic resonance imaging (MRI) is a noninvasive and high-resolution imaging technique widely used to monitor BNNs in the clinic. Through advanced methods like functional MRI (fMRI) and diffusion tensor imaging (DTI), MRI allows for visualization and mapping of the functional connectivity and structural pathways within the brain [55]. This technique is instrumental in understanding the dynamics of brain activity, identifying abnormalities in neural networks, and investigating the impact of neurological disorders. Based on a neural network-based approach, fMRI was used to extract the volume and structural features of cerebral organoids [56]. By using 3D U-Net, a type of convolutional neural network particularly well suited for processing volumetric data, the mean Dice score reached 0.92 ± 0.06 for cerebral organoid segmentation. This highly reliable automated analysis will serve as a powerful tool for comparing healthy organoids with disease organoids characterized by altered growth rates, such as those associated with Zika virus infection or neurodevelopmental disorders that result in microcephaly [26,57,58].

In summary, the two-photon microscopy-based calcium indicator imaging and the MEA recordings are two main prominent techniques used for the functional characterization of ONNs, each with distinct benefits and limitations (Table). Calcium imaging provides high spatial resolution and allows detailed visualization of individual neurons and their networks. These benefits are essential for understanding neuron–neuron or neuron–glial interactions. However, its temporal resolution is lower compared to the MEA method, as calcium signals are indirect measures of neuronal activity. Additionally, calcium imaging involves a complex setup and has the potential for phototoxicity due to prolonged light exposure. By integrating the axially elongated line-confocal illumination with the rolling shutter in scanning light-field microscopy (sLFM), the emerging confocal scanning light-field microscopy (csLFM) achieves high-resolution imaging with higher speed and lower phototoxicity compared with 2-photon microscopy [59]. Conversely, MEA recording provides high temporal resolution, capturing rapid neuronal firing with precision through direct measurement of extracellular electrical signals. It is also suitable for long-term recording from neurons. Nonetheless, the spatial resolution of the common MEA systems is still low, making it challenging to pinpoint specific neuronal activity precisely in brain organoids. Both methods offer complementary insights, while calcium imaging is good for spatial details and the MEA method is good for temporal precision.

**Table.** Comparison of calcium imaging and MEA recording

	Benefits	Challenges
Calcium imaging	High spatial resolution	Low temporal resolution complex
(e.g., 2-photon microscopy)	Deep tissue penetration	Indirect measurement
	Detailed visualization of individual neurons and their networks	Phototoxicity
		Setup
MEA recording	High temporal resolution	Low spatial resolution
	Direct measurement	Recording of surface neurons (or limited depth)
		Long-term recording (e.g., months)

## Dysfunction of Brain Organoid Neural Networks in Neurological Diseases

Several studies have used the anatomical and cytoarchitectural of human brain organoids to model diseases that impact human brain growth or organization, such as microcephaly [26], Huntington’s disease [60], and lissencephaly [61]. However, not only the anatomical structure but also BNNs play a crucial role in the complex functions of the human brain. Indeed, ONNs undergo notable changes in neurological diseases, affecting both their structural and functional integrity. These changes can manifest as alterations in synaptic connectivity, neuron loss, and disrupted communication pathways, reflecting abnormalities of the neuroelectrical activity [62–65].

### Rett syndrome

**Fig. 4.**
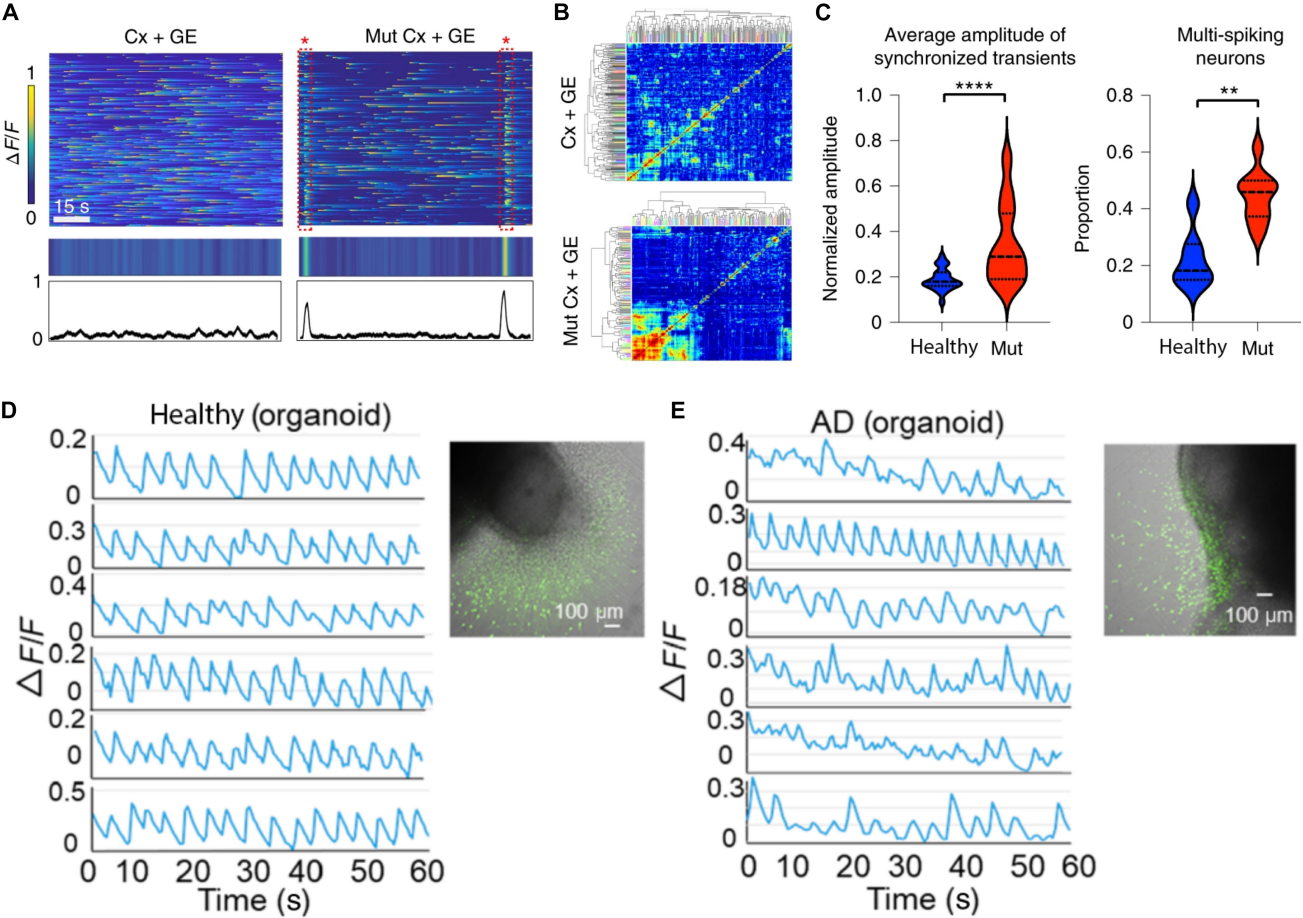
Applications of brain organoid neural networks in neurological diseases. (A) *MECP2*-mutant cerebral cortex–ganglionic eminence fusion organoids exhibit spontaneous synchronized calcium transients that are not observed in the cerebral cortex–ganglionic eminence fusion organoids, reflected in the raw Δ*F*/*F* colorized amplitude plot (top) and synchronization amplitude plot (bottom) [45]. (B) Cluster grams following hierarchical clustering of calcium spiking data [45]. (C) Comparison of the average amplitude of synchronized transients and proportion of multi-spiking neurons [45]. (D) Single-cell tracings of spontaneous calcium surges in the migrated neurons from healthy organoids [71]. (E) Single-cell tracings of spontaneous calcium surges in the migrated neurons from AD organoids [71].

Rett syndrome is a rare genetic neurological disorder caused by mutations in the X-linked gene *MECP2* [66–68]. This disease leads to severe cognitive and physical impairment. Rett syndrome patients often exhibit increased slow wave activity (theta and delta waves), indicating the dysfunction of the prefrontal cortex. Meanwhile, a considerable proportion of individuals with Rett syndrome experience seizures, which are reflected as epileptiform discharges. Consistent with the clinical data, *MECP2*-mutant cerebral cortex–ganglionic eminence fusion organoids exhibited spontaneously synchronized calcium transients across neurons with high amplitude, which is a hallmark of epileptiform discharges (Fig. 4A to C). In contrast, cerebral cortex–*MECP2*-mutant ganglionic eminence fusion organoids did not show any evidence of hypersynchrony, indicating that the mutations in the *MECP2* gene would cause the dysfunction of the GABAergic neurons in cerebral cortex organoids and further disrupt the balance between excitation and inhibition in neural circuits [45]. While the *MECP2*-mutant organoids perform epileptiform-like activity, infants with Rett syndrome often have normal early development for the first 6 to 18 months with no notable changes in neuroelectrical activity. The limitation of human brain organoids in maturity and function connectivity needs to be considered. Moreover, there are difficulties in maintaining organoids for extended periods to model chronic aspects of Rett syndrome.

### Alzheimer’s disease

*PSEN2* is one of the genes known to be implicated in the familial form of Alzheimer’s disease (AD) [69,70]. The gene encodes a protein involved in the production of amyloid-β peptides, which are associated with AD. Individuals with *PSEN2* mutations typically exhibit a marked reduction in the synchronization of neural oscillations across different brain regions, leading to symptoms of dementia, including memory loss and cognitive decline. Similarly, the healthy cerebral organoids displayed synchronized calcium transients, while the calcium transients from AD organoids lacked synchronization (Fig. 4D and E) [71]. AD is an age-related disorder, and current brain organoids lack the ability to model the aging process. Aging-related neural network changes, such as neuronal loss and atrophy, reduced synaptic density, and decline in synaptic plasticity, are critical for studying AD pathogenesis but are not adequately represented in organoids [72,73].

## Perspectives and Conclusions

At present, ONNs present limitations that impede their effectiveness in fully replicating the function of BNNs in humans. Human brain organoids typically lack a vascular system, which is indispensable for ONN function. The vascular system provides the necessary oxygen, nutrients, and waste removal that support high metabolic demands and synaptic activity. Although endothelial cells are exogenously introduced into human brain organoids, the formed rudimentary vessel-like structure cannot improve long-term culture due to lack of function [74]. Future works can focus on constructing human brain organoids with functional vascular networks, from both engineering and developmental biology perspectives. Brain organoids can be combined with microfluidic technology to create more complex and physiologically relevant microenvironments [75–80]. Brain organoids with integrated vascular channels can be cultured under controlled conditions that simulate blood flow and nutrient exchange [81–83]. In addition, assembling blood vessel organoids and brain organoids is another promising strategy to promote the vascularization of brain organoids [84,85].

In the clinic, neurological disorders often disrupt BNNs between different brain regions. For instance, in AD patients, the progressive loss of neurons and synapses is characterized in brain regions crucial for memory and cognition, such as the hippocampus and prefrontal cortex. In major depression, abnormalities in the limbic system, particularly in the amygdala and hippocampus, are common, affecting emotional processing and memory [86]. Disruptions in prefrontal cortex function also contribute to impaired decision-making and emotional control [87]. In autism, key neural network changes include altered connectivity within the default mode network (DMN), which affects self-referential processing and social cognition. Increased connectivity within local brain regions, such as in the frontal and temporal lobes, is often observed, while long-range connectivity between different brain regions may be reduced, impacting the integration of sensory and social information [88]. However, most current brain organoids fail to recapitulate the neural circuits between different brain regions. Although unguided brain organoids contain various interacting brain regions, the cell proportion, and their spatial organization exhibit substantial heterogeneity and unpredictability. The assembloid is a novel system that involves the assembly of multiple types of brain region-specific organoids to create more complex models of BNNs [89,90]. For example, the cerebral organoids and thalamic organoids were fused to model the thalamocortical circuit [91]. Within this assembloid, cortical and thalamic neurons extended axons and established synaptic connections, reconstituting the reciprocal thalamocortical axonal projections crucial for transmitting sensory and motor information in the human brain. Moreover, the forebrain organoid and midbrain organoid were acoustically assembled to model the development of the human mesocortical pathway [92]. After assembly, the midbrain organoid projected tyrosine hydroxylase neurons toward the forebrain organoid. Meanwhile, the assembloid exhibited increased firing rates and synchrony.

In conclusion, the functional ONNs in brain organoids generated by guided or unguided development methods represent a promising avenue for understanding the complexities of brain development and disease modeling in vitro. Through the recapitulation of key aspects of neurodevelopment, human brain organoids offer a unique platform to investigate the formation and dynamics of BNNs. The characterization methods including calcium imaging techniques and electrophysiological recordings may provide valuable insights into the emergence of functional connectivity and network activity within brain organoids. Furthermore, the application of ONNs has facilitated the exploration of pathophysiological mechanisms underlying various neurological disorders, offering new opportunities for drug discovery and personalized medicine.
